# Evaluation of the bacterial ocular surface microbiome in ophthalmologically normal dogs prior to and following treatment with topical neomycin-polymyxin-bacitracin

**DOI:** 10.1371/journal.pone.0234313

**Published:** 2020-06-09

**Authors:** Callie M. Rogers, Erin M. Scott, Benjamin Sarawichitr, Carolyn Arnold, Jan S. Suchodolski

**Affiliations:** 1 Department of Small Animal Clinical Sciences, College of Veterinary Medicine & Biomedical Sciences, Texas A&M University, College Station, Texas, United States of America; 2 Department of Large Animal Clinical Sciences, College of Veterinary Medicine & Biomedical Sciences, Texas A&M University, College Station, Texas, United States of America; University of Lincoln, UNITED KINGDOM

## Abstract

The ocular surface microbiome of veterinary species has not been thoroughly characterized using molecular-based techniques, such as next generation sequencing (NGS), as the vast majority of studies have utilized traditional culture-based techniques. To date, there is one pilot study evaluating the ocular surface of healthy dogs using NGS. Furthermore, alterations in the ocular surface microbiome over time and after topical antibiotic treatment are unknown. The objectives of this study were to describe the bacterial composition of the ocular surface microbiome in clinically normal dogs, and to determine if microbial community changes occur over time or following topical antibiotic therapy. Topical neomycin-polymyxin-bacitracin ophthalmic ointment was applied to one eye each of 13 adult dogs three times daily for seven days, while contralateral eyes served as untreated controls. The inferior conjunctival fornix of both eyes was sampled via swabbing at baseline prior to antibiotic therapy (day 0), after 1 week of treatment (day 7), and 4 weeks after discontinuing treatment (day 35). Genomic DNA was extracted from the conjunctival swabs and primers targeting the V4 region of bacterial 16S rRNA genes were used to generate amplicon libraries, which were then sequenced on an Illumina platform. Data were analyzed using Quantitative Insights Into Molecular Ecology (QIIME 2.0). At baseline, the most relatively abundant phyla sequenced were Proteobacteria (49.7%), Actinobacteria (25.5%), Firmicutes (12%), Bacteroidetes (7.5%), and Fusobacteria (1.4%). The most common families detected were Pseudomonadaceae (13.2%), Micrococcaceae (12%), Pasteurellaceae (6.9%), Microbacteriaceae (5.2%), Enterobacteriaceae (3.9%), Neisseriaceae (3.5%), and Corynebacteriaceae (3.3%). Alpha and beta diversity measurements did not differ in both control and treatment eyes over time. This report examines the temporal stability of the canine ocular surface microbiome. The major bacterial taxa on the canine ocular surface remained consistent over time and following topical antibiotic therapy.

## Introduction

The ocular surface microbiota consists of microorganisms residing on the corneoconjunctival surface and within the tear film. The canine eye is susceptible to ocular surface diseases such as keratoconjunctivitis sicca and infectious ulcerative keratitis, which are often treated with topical broad-spectrum antibiotics such as neomycin-polymyxin-bacitracin [1–7]. Changes in the composition of the ocular surface microbiota may be associated with ocular surface disease, as evidence suggests these commensal microorganisms protect the eye against the proliferation of opportunistic and pathogenic species [8,9]. In addition, antibiotic usage may negatively impact the structure and stability of microbial communities [10–13].

The vast majority of previous studies describing the bacterial ocular surface communities of healthy dogs used traditional culture-based methods [3,5,7,14–16]. The percent of total positive cultures from healthy canine eyes was low, ranging from 29–45%, with Gram-positive bacteria such as *Staphylococcus*, *Streptococcus*, *Bacillus*, and *Micrococcus* spp. most commonly isolated regardless of geography or climate [3,5,7,14–16]. Gram-negative bacteria were less frequently cultured, with *Pseudomonas*, *Moraxella*, *Acinetobacter*, *Neisseria*, *E*. *coli*, *Klebsiella*, and *Enterococcus* spp. comprising less than 4% of isolates [3,5,7,14–16].

Of the traditional culture-based studies involving the canine eye, few evaluated the effect of seasonality or topical antibiotic use on the frequency or type of bacteria sampled from the ocular surface [4,7,12,13]. The percent of positive bacterial cultures from the canine ocular surface were increased during spring and summer compared to autumn and winter, with July having the highest percent of total positive cultures (60%) [7]. Use of the topical fluoroquinolone antibiotic, ofloxacin, for three weeks following cataract surgery in 16 dogs altered the composition of the bacterial community cultured from their conjunctival surfaces, producing >10 colonies per streak compared to <5 colonies per streak prior to treatment [12]. Additionally, increased colony formation was mirrored by increased resistance to topical fluoroquinolones, with the highest percentage of resistant organisms identified 3 weeks post-operatively [12].

There are limitations to culture-dependent studies as cultivable bacteria only represent a fraction of the organisms present in a community. Molecular-based methods, such as 16S rRNA gene sequencing, allow for a more accurate overview of the diversity of bacterial populations colonizing the ocular surface of humans [17–23], horses [24], cats [25–27] and dogs [28].

In the single previous study describing the ocular surface microbiome in dogs using culture-independent molecular-based methods, samples from both eyes were taken at a solitary time point from 10 research-bred dogs [28]. The most common phyla identified were Firmicutes, Actinobacteria, Proteobacteria, and Bacteroidetes. Additionally, the most commonly cultured bacterial genera from canine eyes, *Staphylococcus*, *Streptococcus*, and *Bacillus* spp., comprised only 2.63% of the bacterial community sequenced from the ocular surface [28]. While this preliminary report provides a detailed description of the organisms present, sampling from a single time point limits the usefulness of data to a sole set of environmental and laboratory conditions that may not be repeatable over time. This is compounded by the usage of subjects from a research colony that share many of the same living and dietary conditions, as well as physical characteristics. Samples collected from the previous study, while relevant to a baseline description of organisms present in canines, may not be clinically applicable to dogs living and interacting in diverse environments such as with privately-owned pets.

This study aimed to describe the bacterial compostition of the ocular surface microbiome in healthy privately-owned dogs, and to determine if microbial community changes occur over time or following topical antibiotic therapy. Continued investigations on the effects of antibiotics and other influences on the ocular suface microbiome are necessary to improve our understanding of ocular diseases in both veterinary and physician ophthalmology.

## Materials and methods

### Participants

The study was approved, with owner consent, by the Texas A&M University Institutional Animal Care and Use Committee (Animal Use Protocol #2018–0079). Thirteen dogs, free of ocular disease, were admitted to the Texas A&M Veterinary Medical Teaching Hospital on an out-patient basis for ocular examination and conjunctival swabbing (Table 1). All dogs in the study were recruited by a university-wide email and owned by faculty, students, and staff of Texas A&M College of Veterinary Medicine & Biomedical Sciences. Dogs 8 and 9 as well as dogs 10 and 11 were respectively housed together. The study was performed in May and June in east-central Texas. Sample size was determined from previously published reports indicating 10–12 animals per control and experimental groups would be adequate [29–31].

**Table 1:** Study population: Signalment and randomization of treated eyes for healthy dogs enrolled in the study.

Dog	Breed	Age (Y)	Sex	Treatment Eye	Control Eye
**1**	Great Dane	4Y	SF	OS	OD
**2**	Labrador Retriever	1Y	M	OS	OD
**3**	Cocker Spaniel Mix	11Y	M	OD	OS
**4**	Bloodhound	5Y	SF	OD	OS
**5**	Fox Terrier	8Y	SF	OS	OD
**6**	Mixed Breed	10Y	SF	OD	OS
**7**	Mixed Breed	3Y	SF	OD	OS
**8**	Labrador Retriever Mix	11Y	CM	OS	OD
**9**	Labrador Retriever Mix	12Y	SF	OS	OD
**10**	Dachshund	4Y	CM	OD	OS
**11**	Red Tick Hound	6Y	SF	OD	OS
**12**	Chow Mix	10Y	SF	OS	OD
**13**	Siberian Husky	2Y	CM	OS	OD

Abbreviations: Y: years, SF: spayed female, CM: castrated male, M: male, OS: left eye, OD: right eye.

### Sample collection

All dogs had a complete ophthalmic examination performed by a board-certified veterinary ophthalmologist (EMS), as previously described [24,27]. A routine minimal ophthalmic database was performed, consisting of Schirmer tear test measurements (Intervet Inc., Summit, NJ), fluorescein staining (Amcon Laboratories Inc., St. Louis, MO), and tonometry (TonoVet, Jorgensen Laboratories, Loveland, CO). Any dog with evidence of ocular disease was excluded from the study.

Baseline conjunctival swab samples were collected after the Schirmer tear test and before tonometry and fluorescein staining in order to prevent sample dilution or contamination, as previously described [24,27]. One drop of 0.5% proparacaine (Bausch & Lomb Inc., Bridgewater, NJ) was placed on the ocular surface of each eye to provide topical anesthesia. The inferior conjunctival fornix of both eyes was sampled with Isohelix buccal swabs (Boca Scientific, Inc. Westwood, MA), as previously described [24,27]. Two swabs were used per eye, and each side of the swab was rubbed in the conjunctival fornix 10 times. Swabs were then collected in DNeasy Powerbead tubes with 750 μl buffer containing guanidine thiocyanate (QIAGEN, Inc., Germantown, MD). One drop of 0.5% proparacaine was placed on an unused swab and inserted into an empty PowerBead tube at each of the three time points to serve as three negative controls and confirm lack of environmental contamination. All samples were immediately stored at 4 degrees C and extractions were performed within 24 hours.

Following the collection of baseline samples, one eye of each dog was randomly selected for treatment with topical neomycin-polymyxin B-bacitracin (Dechra Veterinary Products, Overland Park, KS). Online software (https://www.randomizer.org) was employed to randomize left or right eyes into treatment and control groups for each dog. Owners were instructed to instill 1/4-inch strip of the triple antibiotic ointment directly to the ocular surface of the selected eye of their dog three times daily for 7 days. Treatment sheets with detailed instructions and a checklist for treatment application times were distributed to the owners and returned to the investigators at the end of the study to confirm medication compliance (S1 Checklist). Owners returned to the Veterinary Medical Teaching Hospital with their dogs for repeat conjunctival swabs that occurred at the completion of topical antimicrobial therapy (Day 7), and 4 weeks after discontinuing therapy (Day 35). On day 7, sampling occurred approximately 4 hours following the last instillation of medication.

### DNA extraction and sequencing

Genomic DNA was extracted from conjunctival swabs and three negative controls using a single 100 tube DNeasy Powersoil DNA isolation kit (QIAGEN, Inc., Germantown, MD) according to the manufacturer’s instructions. Sequencing of the 16S rRNA gene V4 variable region was performed at MR DNA Laboratory (www.mrdnalab.com, Shallowater, TX, USA) on an Illumina MiSeq platform (Illumina Inc., San Diego, CA) to construct 2x300 paired-end reads using 515F (5’ -GTGYCAGCMGCCGCGGTAA- 3’) and 806R (5´-GGACTACNVGGGTWTCTAAT- 3´) primers [24,27]. The 16S rRNA gene V4 variable region PCR primers 515/806 with barcode on the forward primer were used in a 35 cycle PCR using the HotStarTaq Plus Master Mix Kit (QIAGEN, Inc., Germantown, MD) under the following conditions: 94°C for 3 minutes, followed by 30–35 cycles of 94°C for 30 seconds, 53°C for 40 seconds and 72°C for 1 minute, after which a final elongation step at 72°C for 5 minutes was performed. After amplification, PCR products were checked in 2% agarose gel to determine the success of amplification and the relative intensity of bands. Multiple samples were pooled together (e.g., 100 samples) in equal proportions based on their molecular weight and DNA concentrations. Pooled samples were purified using calibrated Ampure XP beads. Then the pooled and purified PCR product was used to prepare Illumina Truseq nano DNA library.

### Data analysis

Statistical analysis was performed as previously described [24,27]. Sequences were processed and analyzed using Quantitative Insights Into Microbial Ecology (QIIME2 2018.6) [32]. Raw sequences were demultiplexed and the amplicon sequence variant (ASV) table was created using DADA2 [33]. Prior to downstream analysis, sequences assigned as chloroplast, mitochondria, and low abundance ASVs, containing less than 0.01% of the total reads in the dataset were removed. Data were deposited in the National Center for Biotechnology Information (NCBI) Sequence Read Archive (SRA) under the accession number SRP161472.

Alpha diversity (observed ASVs, Shannon, Chao1) was calculated in QIIME2 and analyzed to compare species richness and evenness between control and treatment eyes at baseline and among control and treatment eyes over time. Data were tested for normality using a Shapiro-Wilk test (JMP Pro 14, SAS, Marlow, Buckinghamshire), and followed a non-normal distribution. Therefore, a non-parametric Wilcoxon matched-pairs signed-ranks test was applied for comparison between treatment and control eyes at baseline (PRISM 7, GraphPad Software Inc., San Diego, CA). A non-parametric Friedman test, followed by a Dunn’s multiple comparison post-test were utilized to assess differences in treatment and control eyes over three time points [34].

Beta diversity was calculated in QIIME2 to compare bacterial community composition between samples, and evaluated with the weighted and unweighted phylogeny-based UniFrac distance metric [35] and visualized using Principal Coordinate Analysis (PCoA) plots. An Analysis of Similarity test (ANOSIM) was performed within PRIMER 6 software (PRIMER-E Ltd. Luton, UK) to assess differences in bacterial community composition between samples, where an R statistic near 1 indicates a difference in composition while a value near 0 indicates no difference in composition. Unweighted Unifrac distances over time in both treatment and control eyes were compared using a two-way repeated measures ANOVA (PRISM 7, GraphPad Software Inc., San Diego, CA).

Differences in bacterial taxa relative abundance between eyes at baseline, and among control and treatment eyes over time, were explored. Data did not meet the assumptions for normality using a Shapiro-Wilk test (JMP Pro 14, SAS, Marlow, Buckinghamshire). Therefore, a non-parametric Mann-Whitney U test was elected for statistical comparison between treatment and control eyes at baseline; and a non-parametric Friedman test was used to assess differences in treatment and control eyes over three time points (PRISM 7, GraphPad Software Inc., San Diego, CA). A Dunn’s multiple comparison post-test was then used to determine which time points were significantly different. P-values were adjusted for multiple comparisons and corrected for false discovery rate [36]. P- and q-values <0.05 were considered statistically significant. Lastly, the association of bacterial taxa abundance with each time point in both treatment and control eyes was analyzed by linear discriminant analysis effect size (LEfSe) using Calypso software [37, 38]. This additional step helps to further detect bacterial organisms and functional characteristics differentially abundant between two or more microbial environments [37].

## Results

### Sequence analysis

A total of three negative controls, collected at each time point, failed to show amplification on PCR, indicating the sampling and DNA extraction processes were not contaminated. Sample controls (unused swabs that were processed after the extraction protocol) were also included in sequencing and did not generate any data. All 78 samples (conjunctival swabs collected from 26 eyes at three time points) were positive for PCR amplification and yielded sufficient quality sequences. A total of 2,574,531 sequences were amplified (Min: 13,338.00; Max: 61,820.00; Median: 31,079.50; Mean: 33,006.81; SD: 11,570.95), and rarified to an even sequencing depth, based on the lowest read depth of samples, to 13,338 sequences per sample (S1 Fig). The relative abundance of bacteria was defined for each individual sample.

### Healthy canine eyes at baseline

#### Species richness and diversity at baseline

**Fig 1:**
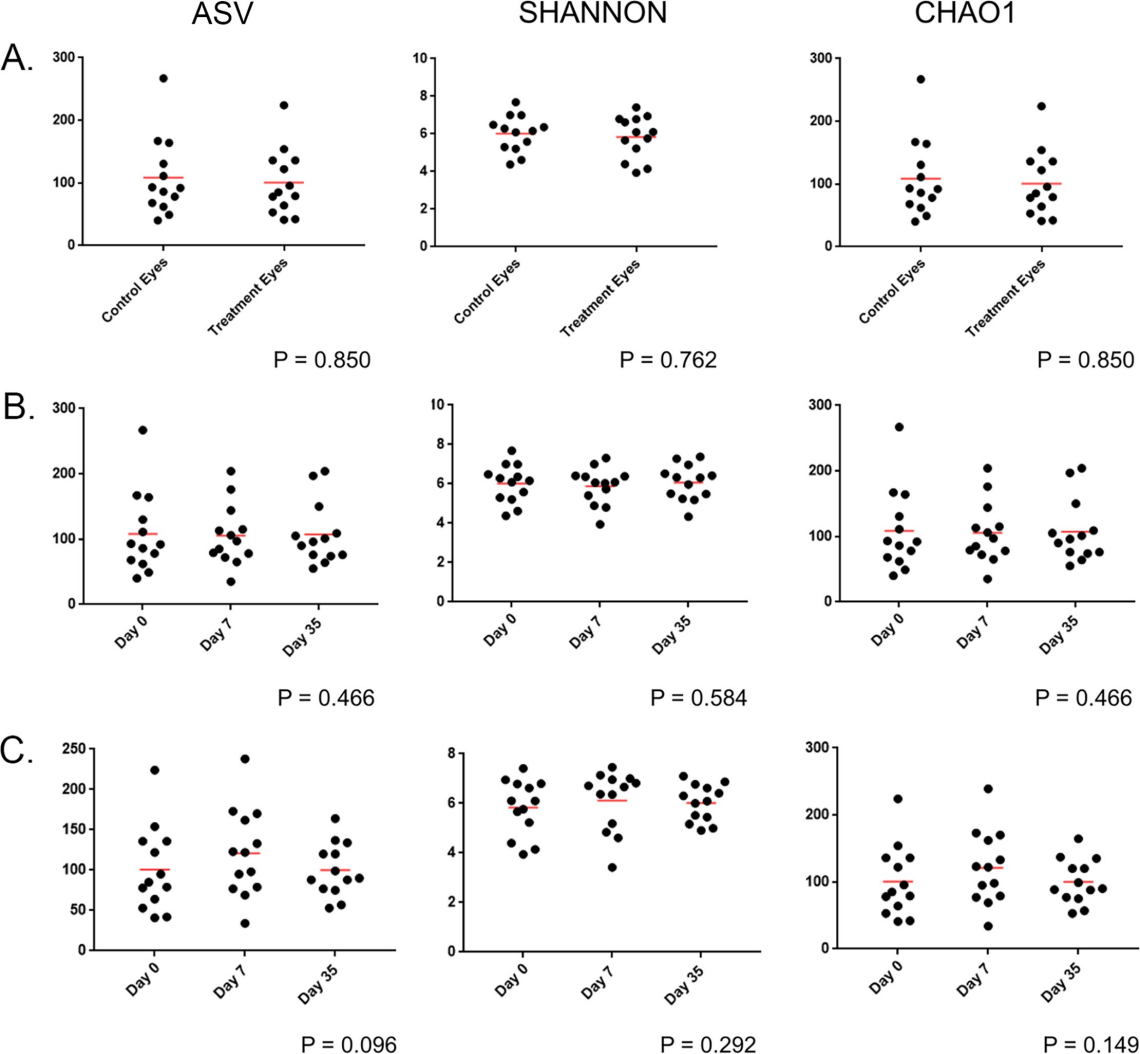
Scatter plots comparing alpha diversity results from (A) treatment and control eyes at baseline (day 0), (B) control eyes of healthy dogs (n = 13) at 3 time points: day 0, day 7, day 35, and (C) treatment eyes of healthy dogs (n = 13) at 3 time points: baseline (day 0), following one week of topical antibiotic therapy (day 7), four weeks after discontinued topical antibiotic therapy (day 35). Each dot corresponds to one eye from 13 healthy dogs. There is no difference in alpha diversity between control and treatment eyes at baseline (Wilcoxon match-pairs signed-ranks test). Alpha diversity did not differ in control eyes or treatment eyes over time (Friedman test and Dunn’s post-test).

Species richness (observed ASVs and Chao1) and abundance and evenness (Shannon) were analyzed to examine taxonomic diversity within a sample. There was no significant difference between control eyes and treatment eyes at baseline (prior to antibiotic treatment) for all three alpha diversity metrics (S1 Table). Hence, eyes at baseline exhibited similar results with regard to species richness, evenness, or abundance (Fig 1A).

#### Microbial community structure at baseline

**Fig 2:**
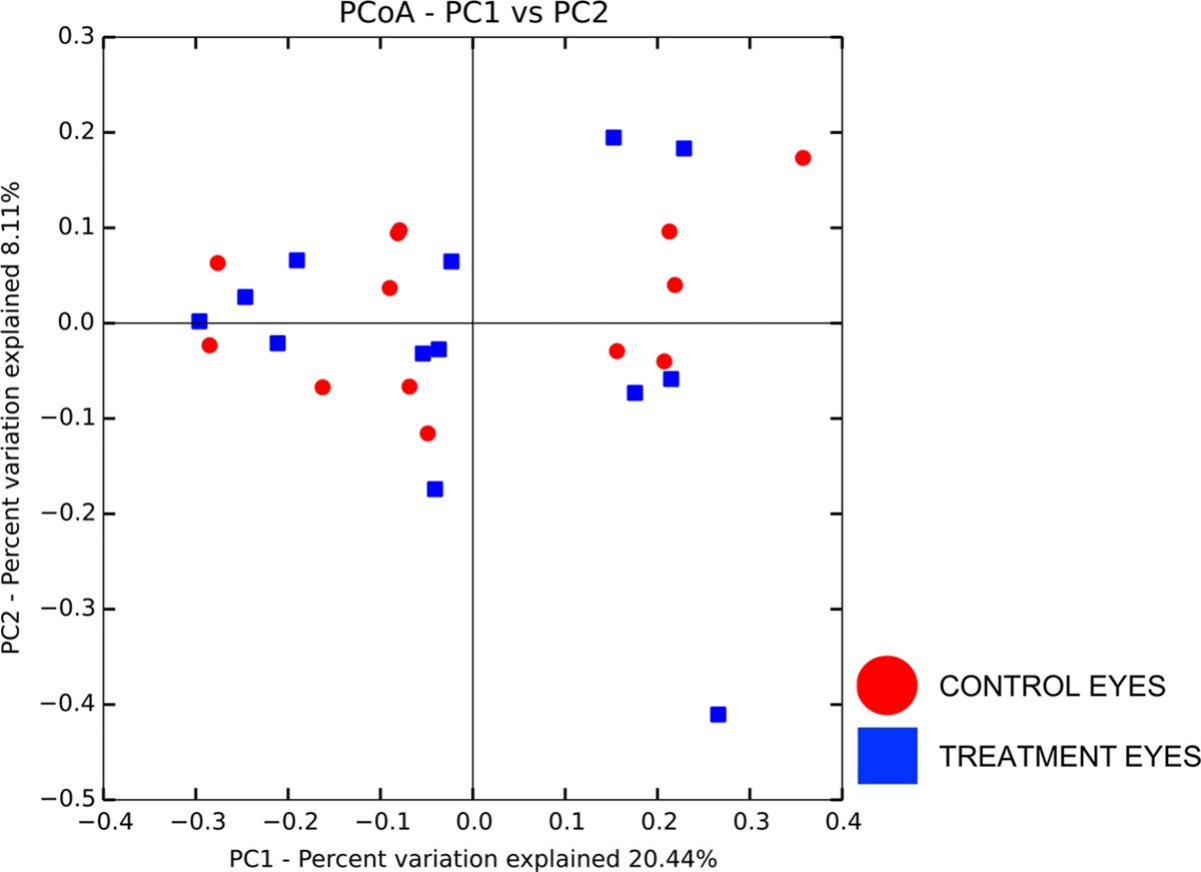
Principal coordinate analysis comparing treatment and control eyes at baseline (day 0). Principal coordinate analysis (PCoA) plot based on the unweighted UniFrac distance metric illustrating no difference in beta diversity by a lack of clustering between eyes at baseline. Each dot corresponds to the microbial composition of one eye.

Beta diversity measures were analyzed to examine taxonomic diversity between samples. Weighted and unweighted UniFrac distance matrices showed no difference in community structure between treatment and control eyes at baseline (R = -0.06, R = -0.05, respectively, p > 0.05), with a lack of clustering observed on principal coordinate analysis plots (PCoA) (Fig 2). Weighted UniFrac PCoA plots gave us similar results (data not shown). Clustering was also not apparent by individual dog or shared housing (S2 Fig and S3 Fig).

#### Microbial community composition at baseline

**Fig 3:**
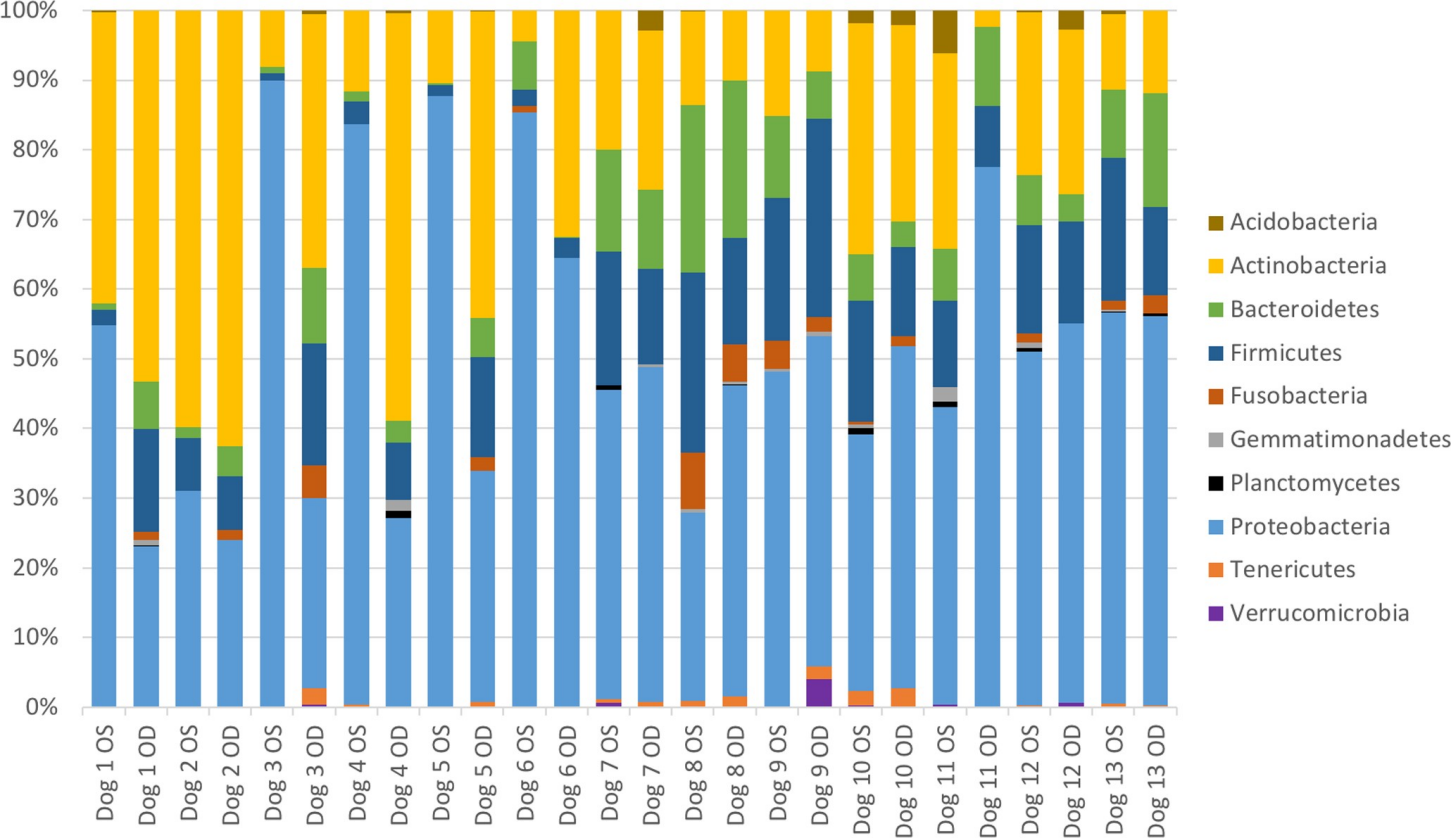
Bacterial phylum composition in healthy dogs. Average relative taxa abundances from the ocular surface of healthy dogs, annotated to the level of bacterial phylum, at baseline (day 0). Each bar chart represents the left (OS) or right (OD) eyes of 13 dogs.

The relative abundance of bacterial taxa did not differ between treatment and control eyes at baseline (Mann-Whitney U test). Results from all sampled eyes were averaged to describe the bacterial composition of the ocular surface from 13 healthy dogs. A total of 10 bacterial phyla were identified, with 4 phyla consistently present in all 26 eyes (S2 Table). The most prevalent phyla detected were Proteobacteria (49.7%), followed by Actinobacteria (25.5%), Firmicutes (12.0%), and Bacteroidetes (7.5%) (S2 Table, Fig 3).

**Fig 4:**
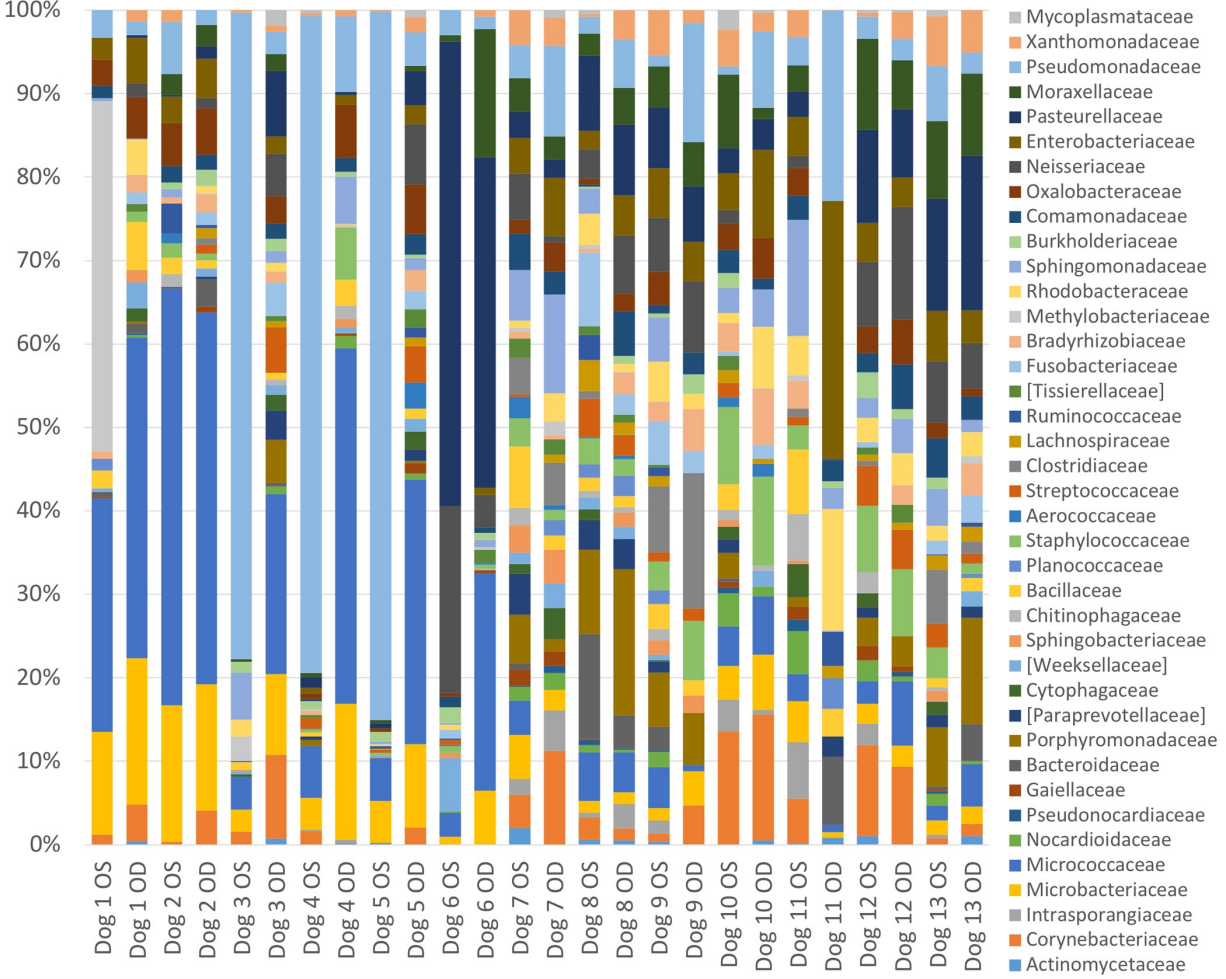
Bacterial family composition in healthy dogs. Average relative taxa abundances from the ocular surface of healthy dogs, annotated to the level of bacterial family, at baseline (day 0). Each bar represents the left (OS) or right (OD) eyes of 13 dogs.

A total of 46 bacterial families were identified at ≥1% relative abundance, and 14 families were present in at least 20/26 eyes. Only two families, Microbacteriaceae and Pseudomonadaceae, were present in all 26 eyes (S2 Table). The most prevalent bacterial families identified were Pseudomonadaceae (13.2%), Micrococcaceae (12.0%), Pasteurellaceae (6.9%), Microbacteriaceae (5.2%), Enterobacteriaceae (3.9%), Neisseriaceae (3.5%), and Corynebacteriaceae (3.3%) (S2 Table, Fig 4). Frequently cultured bacterial families from the ocular surface of healthy dogs, such as Micrococcaceae, Corynebacteriaceae, Staphylococcaceae, Bacillaceae, and Streptococcaceae, represented 12.0%, 3.3%, 2.3%, 1.5% and 1.2% of the bacterial families sequenced, respectively. Individual variation in relative abundances of bacterial taxa was observed both between eyes and between dogs (Figs 3 and 4). Throughout all samples, an average of 107 different ASVs were sequenced.

### Temporal variability in control eyes

In order to investigate temporal stability of the ocular surface microbiome in clinically normal dogs, additional conjunctival swabs were collected from control eyes one week (day 7) and five weeks (day 35) after the initial samples (day 0, baseline).

#### Species richness and diversity over time

Alpha diversity metrics were unchanged in control eyes between the three sampled time points (S3 Table and Fig 1B).

#### Microbial community structure over time

**Fig 5:**
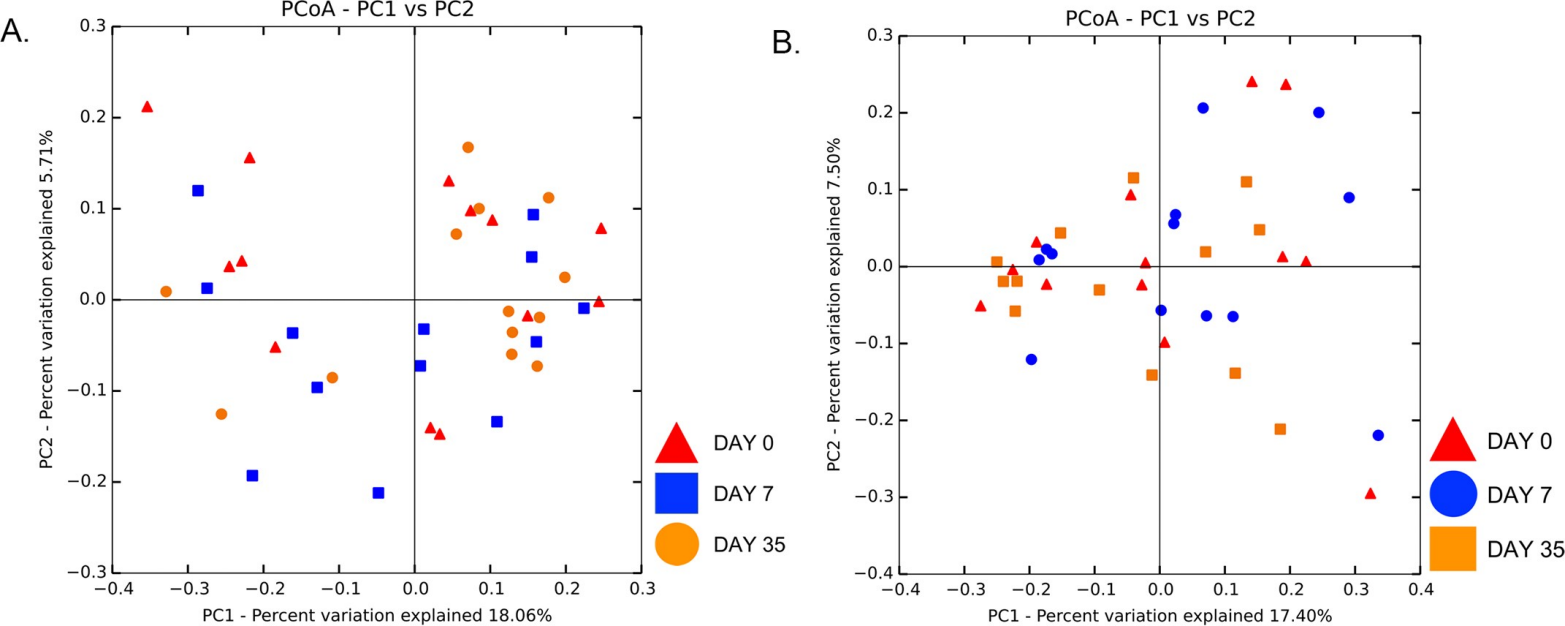
Principal coordinate analysis comparing **(A)** control eyes over time, and **(B)** treatment eyes over time. Principal coordinate analysis (PCoA) plot based on the unweighted UniFrac distance metric illustrating no difference in beta diversity by a lack of clustering among **(A)** 13 control eyes and **(B)** 13 treatment eyes at three time points: day 0, day 7, day 35. Each dot corresponds to the microbial composition of one eye.

**Fig 6:**
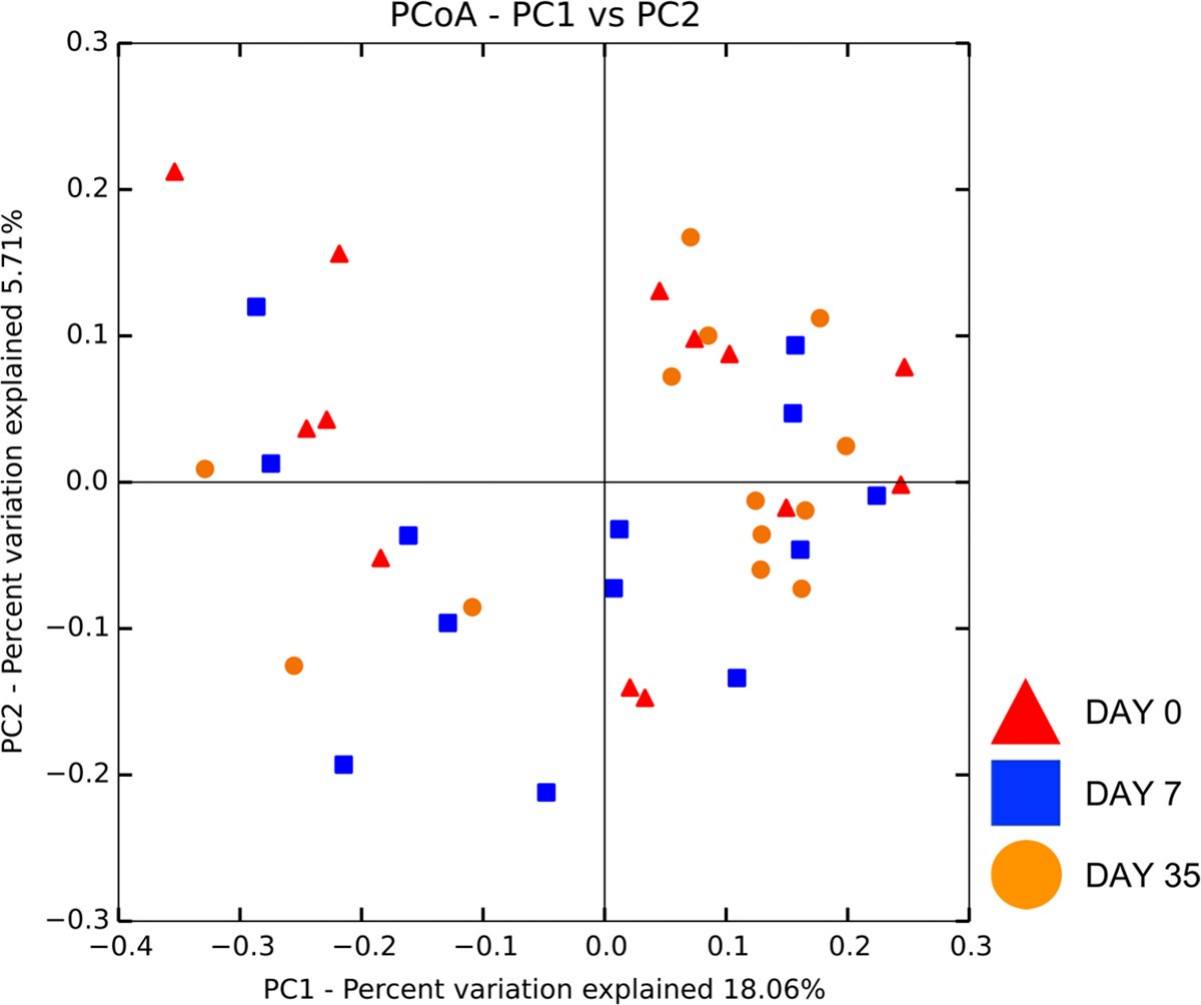
Unweighted Unifrac distances in control eyes (n = 13) and treatment eyes (n = 13) over time. There is no significant difference in Unifrac distances measured from baseline (day 0), suggesting bacterial communities remain stable over time (Two-way ANOVA; p > 0.05).

There was no significant change in beta diversity associated with time, evidenced by the lack of clustering in PCoA plots (Fig 5A). The global community structure of control eyes did not vary over time based on ANOSIM (weighted UniFrac, R = 0.01, R = 0.06, R = -0.03 for day 0 vs. 7, day 0 vs. 35, and day 7 vs. 35, respectively, p > 0.05); (unweighted UniFrac, R = -0.03, R = -0.003, R = 0.01 for day 0 vs. 7, day 0 vs. 35, and day 7 vs. 35, respectively, p > 0.05). Weighted UniFrac PCoA plots gave us similar results (data not shown). There was no difference in unweighted Unifrac distances over time in control eyes, when measured from baseline (day 0) (p > 0.05) (Fig 6).

#### Microbial community composition over time

**Fig 7:**
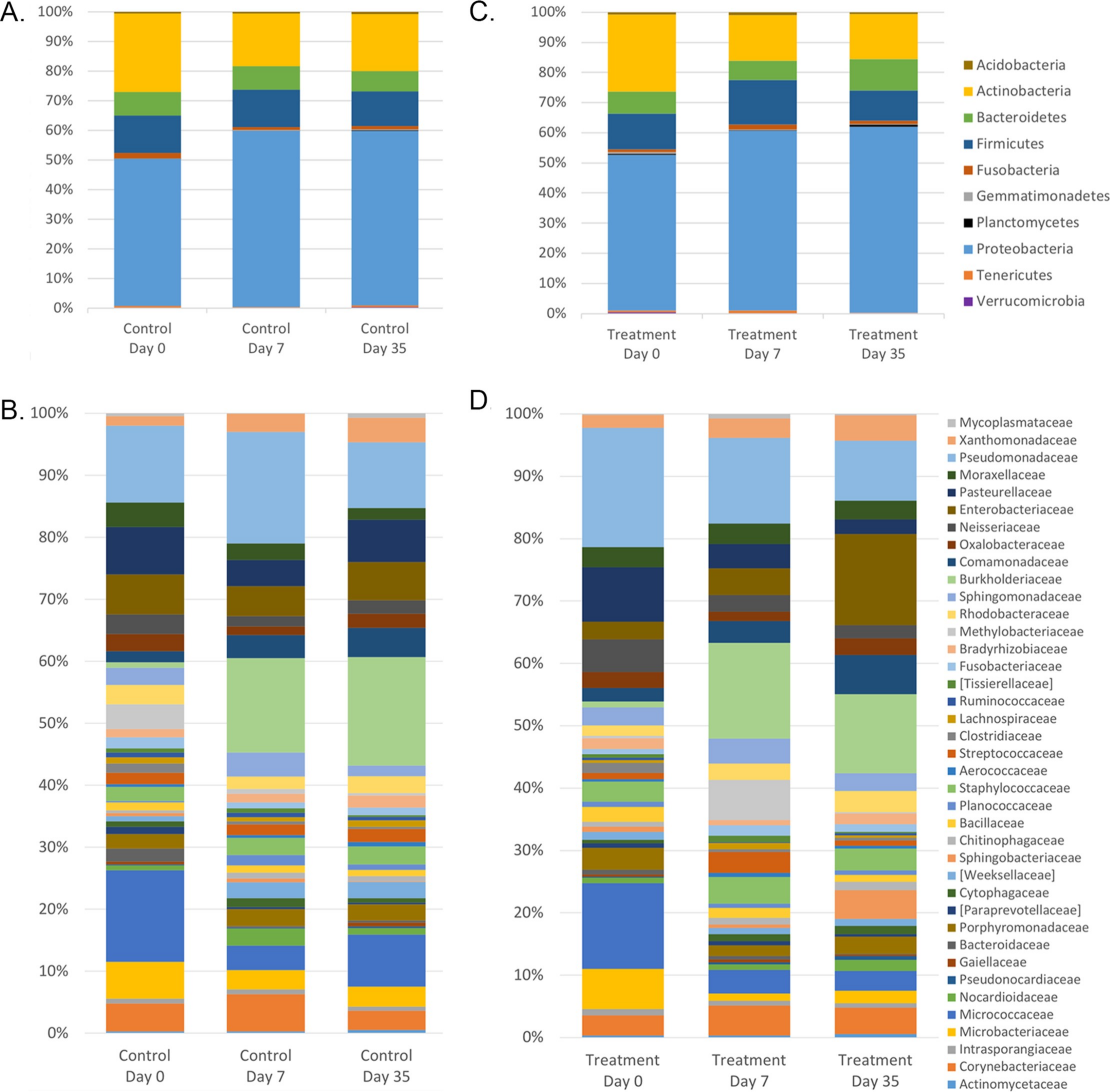
Bacterial phylum and family composition of (A, B) control eyes and (C, D) treatment eyes over time. Bars represent mean percentage of taxa present at ≥ 1% mean relative abundance and totaling 100% at each time point.

Fig 7A and 7B displays the mean relative abundance of bacteria in control eyes at each of the time points sampled. Variance in bacterial abundance was observed between individual dogs (S4 Fig). No significant changes in abundance were detected at the phylum, family, or genus level (q > 0.05) (Table 2).

**Table 2:** Temporal variation in the relative abundance of bacterial taxa sequenced from the canine ocular surface of healthy control eyes. Median percentages and ranges of relative bacterial abundance, annotated to the level of family and genus with p-values <0.05 are shown. Phylum data are included for comparison.

Taxon	Day 0		Day 7		Day 35			
Phylum	Median %	Range %	Median %	Range %	Median %	Range %	P-value *	Q-value **
Family								
*Genus*								
**Proteobacteria**	48.7	23.7–83.2	55.5	33.1–96.3	54.2	39.9–87.7	0.125	0.871
Oxalobacteraceae	2.6^a^	0–5.6	0.6^b^	0–6.4	1.7^a,b^	0–5.4	0.036	0.364
*Burkholderia*	0^a^	0–1.0	0.7^b^	0–61.7	0.9^b^	0–75.8	0.003	0.154
*Delftia*	1.3^a^	0–2.5	1.0^a^	0–4.6	1.2^a^	0–3.9	0.019	0.311
*Unclassified Neisseriaceae*	0^a^	0–3.8	0^a^	0–5.7	0^a^	0–2.5	0.039	0.311
**Actinobacteria**	23.3	2.3–61.9	15.8	2.3–40.0	18.0	6.3–48.0	0.368	0.871
Unclassified Actinomycetales	0^a^	0–0.2	0^a,b^	0–0.9	0.4^b^	0–1.3	0.006	0.145
*Corynebacterium*	1.9^a^	0–13	2.8^a,b^	0.4–25.3	3^b^	0–6.9	0.006	0.154
**Firmicutes**	13.8	2.1–24.3	10.0	1.1–23.8	11.6	2.0–23.1	0.926	0.926
Planococcaceae	0^a^	0–1.7	1.2^b^	0–6.2	0^a,b^	0–4.0	0.003	0.145
**Bacteroidetes**	7.1	0.2–22.6	7.2	0.2–22.2	6.4	0.1–16.7	0.926	0.926
**Tenericutes**	0.3	0–2.7	0	0–1.3	0	0–4.4	0.397	0.871
Mycoplasmataceae	0.3^a^	0–1.5	0^a^	0–0.5	0^a,b^	0–4.4	0.034	0.364

Median values not sharing a common superscript differ significantly (p < 0.05, Dunn’s multiple comparison post-test).

*: P-values based on the Friedman test

**: Q-values adjusted based on the Benjamini & Hochberg False discovery rate

Similar to the trends reported in Table 2, LEfSe analysis indicated that *Burkholderia* and unclassified Actinomycetales were increased on day 35 among control eyes in comparison to day 0 (baseline) and day 7 (Table 3). LEfSe demonstrated additional changes in relative taxa abundance of few bacterial families and genera over time (Table 3).

**Table 3:** Linear discriminant analysis of bacterial taxa in control eyes and their associations with the sampling time point. LDA scores >3.0 are included.

Taxa	LDA score	Time point
**Family**		
Paraprevotellaceae	3.66	Day 0
Comamonadaceae	4.12	Day 35
Unclassified Actinomycetales	3.62	Day 35
***Genus***		
*Ureaplasma*	3.86	Day 0
*P75a5*	3.97	Day 0
*Salinibacterium*	4.29	Day 0
*Ralstonia*	3.68	Day 35
*Unclassified Actinomycetales*	4.03	Day 35
*Burkholderia*	4.84	Day 35

### Temporal variability in eyes treated with neomycin-polymyxin-bacitracin

In order to evaluate the temporal stability of the ocular surface microbiome in clinically normal dogs following the application of topical antibiotics, additional conjunctival swabs were collected from treatment eyes subsequent to the baseline samples (day 0). Swabbing was repeated after eyes were treated three times daily for one week (day 7), and four weeks after the completion of antibiotic therapy (day 35).

#### Species richness and diversity over time

Alpha diversity metrics were unchanged in treatment eyes between the three sampled time points (S3 Table and Fig 1C).

#### Microbial community structure over time

There was no significant change in beta diversity associated with time, evidenced by the lack of clustering in PCoA plots (Fig 5B). The global community structure of treatment eyes did not vary over time based on ANOSIM (weighted UniFrac, R = -0.004, R = 0.11, R = 0.02 for day 0 vs. 7, day 0 vs. 35, and day 7 vs. 35, respectively, p > 0.05); (unweighted UniFrac, R = -0.01, R = -0.02, R = -0.005 for day 0 vs. 7, day 0 vs. 35, and day 7 vs. 35, respectively, p > 0.05). Weighted UniFrac PCoA plots gave us similar results (data not shown). There was no difference in unweighted Unifrac distances over time in treatment eyes, when measured from baseline (day 0) (p > 0.05) (Fig 6). Therefore, bacterial communities on day 7 compared to day 0 were no more different than communities at day 35 compared to day 0. Additionally, Unifrac distances between control eyes and treatment eyes did not vary, suggesting bacterial communities remained similar over time regardless of treatment (p > 0.05) (Fig 6).

#### Microbial community composition over time

Fig 7B and 7C illustrates the mean relative abundance of bacteria in treatment eyes at each of the time points sampled. Variance in bacterial abundance was observed between individual dogs (S5 Fig). Friedman and Dunn’s multiple comparison tests revealed the abundance of some bacterial families and genera changed on the ocular surface of treatment eyes over time (q < 0.05) (Table 4). No significant changes were detected at the phylum level (q > 0.05). At the family level, treatment eyes had significantly more Microbacteriaceae on day 0 compared to day 7 and day 35 (p = 0.001, q = 0.029). At the genus level, treatment eyes had significantly more *Salinibacterium* on day 0 compared to day 7 and day 35 (p = 0.001, q = 0.032). Unclassified Enterobacteriaceae were significantly enriched in treatment eyes on day 35, compared to day 0 and day 7 (p = 0.001, q = 0.040).

**Table 4:** Temporal variation in the relative abundance of bacterial taxa sequenced from the canine ocular surface of treatment eyes. Median percentages and ranges of relative bacterial abundance, annotated to the level of family and genus with p-values < 0.05 are shown. Phylum data are included for comparison.

Taxon	Day 0		Day 7		Day 35			
Phylum	Median %	Range %	Median %	Range %	Median %	Range %	P-value *	Q-value **
Family								
*Genus*								
**Proteobacteria**	43.2	22.5–89.9	54.8	37.8–97.5	63.6	37.0–86.5	0.199	0.994
Methylobacteriaceae	0.1^a^	0–2.9	1.0^a^	0–56.8	0.1^a^	0–0.8	0.027	0.442
Enterobacteriaceae	2.9^a^	0–4.5	3.9^a,b^	0–6.4	9.0^b^	2.2–61.1	0.012	0.312
*Unclassified Enterobacteriaceae*	2.3^a^	0–4.5	1.8^a^	0–6.4	7.1^b^	2.2–60.9	**0.001**	**0.040**
*Unclassified Sphingomonadaceae*	0^a^	0–0.9	0^a^	0–1.5	0^a^	0–0	0.018	0.186
*Delftia*	0^a^	0–2.1	1.2^a,b^	0–3.3	2.1^b^	0–8.2	0.012	0.186
*Stenotrophomona*	0.2^a^	0–2.2	0.8^a,b^	0–5.9	1.0^b^	0–11.3	0.016	0.186
**Actinobacteria**	19.4	4.4–59.8	13.0	1.4–29.0	13.5	3.9–22.6	0.368	0.920
Microbacteriaceae	3.0^a^	1.0–14.7	0.7^b^	0–2.2	1.2^b^	0–7.2	**0.001**	**0.029**
*Salinibacterium*	2.3^a^	1.0–14.7	0.4^b^	0–2.2	0.3^b^	0–7.2	**0.001**	**0.032**
Micrococcaceae	3.8^a^	0.6–44.8	2.8^a,b^	0.4–11.3	1.7^b^	0–8.8	0.037	0.442
**Firmicutes**	12.2	1.1–27.6	15.5	0.5–28.2	10.9	2.9–15.6	0.794	0.992
**Bacteroidetes**	6.6	0.3–20.6	6.4	0.2–15.1	7.2	0–36.4	0.926	0.926

Median values not sharing a common superscript differ significantly (p < 0.05, Dunn’s multiple comparison post-test).

*: P-values based on the Friedman test

**: Q-values adjusted based on the Benjamini & Hochberg False discovery rate

LEfSe analysis exhibited similar temporal changes in relative taxa abundance of some bacterial families and genera (Table 5). Concurrent with Table 4, Microbacteriaceae and *Salinibacterium* were comparatively increased among treatment eyes on day 0 (baseline) compared to day 7 and day 35, while unclassified Enterobacteriaceae were enriched on day 35 compared to day 0 and day 7 (Table 5 and Fig 7C and 7D).

**Table 5:** Linear discriminant analysis of bacterial genera in treatment eyes and their associations with the sampling time point. LDA scores >3.0 are shown.

Taxa	LDA score	Time point
**Family**		
Microbacteriaceae	4.49	Day 0
Micrococcaceae	4.75	Day 0
Xenococcaceae	4.36	Day 7
Methylobacteriaceae	4.47	Day 7
Enterobacteriaceae	4.70	Day 35
***Genus***		
*Salinibacterium*	4.74	Day 0
*Pseudomonas*	4.69	Day 35
*Unclassified Enterobacteriaceae*	4.88	Day 35

## Discussion

The bacterial community associated with the canine ocular surface is more complex and diverse than previously reported with culture-dependent studies. All 26 eyes sampled contained bacteria from at least 4 phyla and 2 families at >1% relative abundance (S2 Table). Furthermore, the ocular surface microbiome in our population of privately-owned dogs is distinct to the eye when compared to previous reports on the canine skin, mouth, and nasal mucosa [39–41].

The most common bacterial phyla and their relative proportions detected on the canine ocular surface in the present study, Proteobacteria (49.7%), Actinobacteria (25.5%), Firmicutes (12.0%), and Bacteroidetes (7.5%), are analogous to descriptions of the human [17,22,23] and equine [24] ocular surface microbiome. Preliminary studies examining the ocular surface microbiome of cats and dogs using culture-independent methods identified the same bacterial phyla at different proportions, with a relatively increased abundance of Firmicutes across all feline (30–43%) [25,27] and canine samples (34.9%) [28]. It is important to note; however, that limitations exist when comparing microbiome studies as numerous variations in methodologies for DNA extraction, sequencing, analysis, contaminant filtering, and clustering strategies may influence the results.

The most common bacterial families and their relative proportions sequenced from the majority of canine eyes sampled were Pseudomonadaceae (13.2%), Micrococcaceae (12.0%), Pasteurellaceae (6.9%), Microbacteriaceae (5.2%), Enterobacteriaceae (3.9%), Neisseriaceae (3.5%), and Corynebacteriaceae (3.3%) (Fig 4). Gram-positive bacteria regularly cultured from the canine ocular surface, such as Micrococcaceae, Corynebacteriaceae, Staphylococcaceae, Bacillaceae, and Streptococcaceae, represented 12.0%, 3.3%, 2.3%, 1.5% and 1.2% of the bacterial families sequenced, respectively, and were detected in 65–96% of the eyes sampled (S2 Table). A larger proportion of the most abundantly sequenced microorganisms were Gram-negative, contradicting the previous culture-based evidence of a primarly Gram-positive ocular microbiota. This finding may be explained by inherent bias of culture-based detection toward fast growing bacteria that can be easily cultivated in standard laboratory settings [17]; however, additional NGS studies with appropriate sequencing of negative controls and rigourous contaminant filtering are necessary to validate our findings.

This study identified numerous taxa that were never before linked to the canine ocular surface, possibly due to lack of cultivability. Previously unrecognized organisms that may inhabit the canine ocular surface include families from 4 phyla: Proteobacteria (Sphingomonadaceae, Oxalobacteraceae, Unclassified Oxalobacteraceae, Rhodobacteraceae, Methylobacteriaceae, Xanthomonadeceae, Unclassified Xanthomonadeceae, Bradyrhizobiaceae, Unclassified Bradyrhizobiaceae, Burkholderiaceae, Comamonadaceae), Actinobacteria (Unclassified Micrococcaceae, Intrasporangiaceae), Firmicutes (Lachnospiraceae) and Bacteroidetes (Cytophagaceae, Sphingobacteriaceae, Weeksellaceae) (S2 Table). Although NGS has allowed us to detect the presence of these taxa on the canine ocular surface, their role in the microbiome and impact on the health of the canine eye is currently unknown.

In control eyes, the relative abundances of bacterial taxa, along with alpha and beta diversity metrics, did not vary when compared over three separate time points: day 0, day 7, and day 35. This finding supports the notion that species richness, community structure, and community composition of the canine ocular surface microbiome are not significantly altered over time.

The ocular surface, similar to the skin, mouth, and nasal cavity, is an open and exposed system in constant contact with its environment and external microbes. However, the ocular surface is thought to have a relatively low microbial abundance compared to other open systems in the body [18,23], primarily due to innate defense mechanisms that protect against infection such as blinking, tearing and the presence of antimicrobial secretions [42]. One may argue that such mechanisms prevent the development of a stable core bacterial population, only allowing for a haphazard collection of transient organisms to temporarily reside on the ocular surface. Individual variation in the relative abundances of taxa both between eyes and between dogs at baseline were observed at the family level (S4 and S5 Figs). This finding; however, is not unique to the ocular surface, as a high degree of interindividual variability exists within human and animal microbiomes, and is likely attributed to environmental factors and host genetics [23]. A shift in bacterial communities; however, was not measured over time or between treatment and control eyes in our study, suggesting the general bacterial composition of the ocular surface remains relatively unchanged (and is not a haphazard collection) despite this variability (Fig 6). This study, therefore, proposes the presence of both a core and transient microbial population on the canine ocular surface. Similar to the equine [24] and feline [27] eye, several bacteria taxa were present in the majority of canine eyes at each time point sampled, consistent with the presence of a stable core community of microbes. Many of the bacterial families listed in Fig 7B likely constitute the core microbiome of the canine ocular surface; however, additional longitudinal investigations are necessary with more rigorous contaminant filtering to support our findings and define its composition.

In treatment eyes, alpha and beta diversity metrics did not vary when compared between three separate time points: prior to antibiotic therapy (day 0), following one week of treatment with neomycin-polymyxin-bacitracin (day 7), and four weeks after stopping antibiotic therapy (day 35). As noted in control eyes, there were no significant differences in the relative abundance of bacterial phyla in treatment eyes over time. Although the current study detected minor fluctuations in the relative abundance of few bacterial families and genera in treatment eyes over time (Table 4 and Table 5), comparable trends were noted in control eyes. We suspect these minor variations were more likely caused by a combination of host and environmental factors rather than antibiotic treatment. Similar studies of the equine and feline ocular surface also recognized a stable bacterial community that remained consistent following short-term topical broad-spectrum antibiotic use [24,27]. This demonstrates the bacterial microbiome of the ocular surface is not altered by the short term use of broad spectrum topical antibiotics, as species richeness, community structure, and global community composition remained stable.

Neomycin-polymyxin-bacitracin is a broad-spectrum triple antibiotic ointment commonly prescribed in veterinary medicine to prevent or treat bacterial infections of the ocular surface. Neomycin disrupts bacterial protein synthesis, while polymyxin B increases the permeability of bacterial cell membranes [43]. Both antibiotics provide a Gram-negative spectrum of activity [4,43]. Bacitracin inhibits bacterial cell wall synthesis and is primarily active against Gram-positive bacteria [4,43]. This combination of antibiotics is most effective for surface disease, as transcorneal and ocular penetration of the drugs are poor [44]. Additionally, the bioavailability of topically applied medication is generally low [45]. Therefore, systemic absorption of neomycin-polymycin-bacitracin is not expected to occur nor reach therapeutic concentrations on the ocular surface of the contralateral (control) eye. However, in the absence of data showing antibiotic levels in both eyes or longitudinal data in completely treatment-naïve dogs, we cannot completely rule out the possibility of systemic absorption contributing to the similar results in both treatment and control eyes.

There are several reasons neomycin-polymyxin-bacitracin did not shift the ocular surface microbiome as we often see when profiling the gastrointestinal microbiome following parenteral antibiotics. For one, topically applied ophthalmic drugs have a limited retention time on the ocular surface due to lacrimation and drainage. After application to the eye, ointment is retained in the conjunctival fornix until the petrolatum base melts, exposing and delivering the drug to the tear film [46]. Although ointments have increased retention times compared to aqueous solutions and suspensions, the continual turnover of tears will eventually eliminate the medication in 2–3 hours [47]. Therefore, with three times daily application, a common dosage frequency for ophthalmic ointments, the regional microbiome of healthy eyes may be able to rebound and repopulate between dosages. This broad-spectrum combination of antibiotics is frequently used in veterinary medicine as prophylactic therapy to prevent infection by opportunistic bacteria following corneal ulceration, and to treat conjunctival bacterial overgrowth in patients with ocular surface disease such as dry eye [1–7]. While effective in these scenarios, more intensive antibiotic therapy is required with the treatment of bacterial keratitis. Dogs, especially brachycephalic breeds, are susceptible to severe ocular surface diseases that require prolonged antibiotic therapies for several weeks to months [1–7]. Chronic and frequent use of ophthalmic antibiotics of several week duration may have more notable effects on the ocular surface microbiota of diseased eyes, and enable the development of antibiotic resistence [10–13]. Therefore, additional studies are warranted to further evaluate chronic topical antimicrobial use and its impact on the canine ocular surface microbiome.

This study evaluated a relatively small and heterogeneous canine population, representing a diverse sampling of privately-owned dogs with different housing environments. Although homogeneity is recommended when targeting a study population to eliminate compounding variables, our findings are more likely to represent what is found in the general canine population compared to a research colony of dogs. Although two pairs of dogs shared the same household in our study, their samples did not display apparent clustering on principal coordinate analysis plots (S3 Fig), nor did we find clustering by eyes of individual dogs (S2 Fig). Currently, the effect of population variations such as age, sex, season, geography, and other environmental factors on the composition of the ocular surface microbiome of veterinary species is unknown. Future investigations incorporating larger and more geographically diverse canine populations are warranted to limit bias and more accurately describe the variability present on the ocular surface microbiome.

In addition to a relatively small sample size, owner compliance was a potential limitation to this study. All dogs were owned by personnel of the Veterinary Medical Teaching Hospital whose training and medical skills allowed for the appropriate administration of ophthalmic ointment to the eye. While treatment sheets with detailed instructions and a checklist for treatment application times were distributed to the owners and returned to the investigators at the conclusion of the study, compliance was not verified further.

Additional limitations are intrinsic to the interpretation of microbiome studies, including the assessment of relative abundance, which does not take into account the absolute bacterial quantities present in a sample. The microbiome datasets generated from high-throughput sequencing are compositional, yet many microbiome analyses, including those in this study, use non-compositional models where an increase in proportion does not always translate into an increase in absolute abundance of bacteria [48]. Quantitative PCR of known, previously identified organisms is required to detect absolute quantities and should be considered, along with the compositional analyses of microbiome datasets, with future NGS studies. The viability of organisms detected via NGS cannot be determined, possibly leading to an overrepresentation of nonviable organisms controlled by the host’s immune response [18].

Relatively low biomass environments, such the ocular surface, are more susceptible to having contaminating DNA from laboratory reagents negatively affect the results, and some bacteria noted in this study have been reported as common contaminants [49]. Although DNA extraction and sequencing of our sample collection blanks (unused swabs) was performed and did not yield any data, a limitation to this study is the failure to sequence DNA extraction blanks (reagents only with no swab included). The sequencing laboratory in our study reported the likelihood of background noise interfering with the sample data to be remote at best. However, a lack of subtractive filtering to remove contaminants, even when negative sample controls fail to show amplification on PCR or data on sequencing, may still allow potential reagent contaminants to confound the interpretation of microbiome data [50]. This limitation may impact the reliability of our results and other studies that failed to sequence extraction and PCR reagents blanks. Future studies will include sequencing of our negative sample and reagents controls, regardless of PCR amplification, to eliminate any concerns for contamination.

The usage of a topical anesthetic, while necessary to apply greater pressure during swabbing to obtain a more accurate sample of the ocular surface [17,21], may also limit microbial diversity by diluting the ocular surface [51]. Despite these inherent limitations, there remains a wealth of valuable information to improve our insight into the ocular surface microbiome and elucidate its influence on the eye.

## Conclusion

This report investigates the temporal stability of the canine ocular surface microbiome in clinically normal eyes with and without topical broad-spectrum antibiotic therapy. In contrast to previous culture-dependent studies, all canine eyes demonstrated the presence of bacteria, several of which were Gram-negative and formerly extraneous to the canine eye. Similar to equine and feline studies using molecular-based techniques, a diverse and stable bacterial community was identified to inhabit the canine ocular surface, and recognized to remain consistent over time and following short-term topical broad-spectrum antibiotic use. Investigations into the canine ocular surface microbiome in diseased eyes are ongoing to reveal if changes to bacterial microbial communites are associated with ocular disease.

## Supporting information

S1 Checklist Example of treatment sheet with instructions and checklist for medication application times distributed to owners for increased compliance. (PDF)

S1 Table Alpha diversity averages for eyes at baseline (day 0) measured at 13,338 sequences per sample. (DOCX)

S2 Table Taxa present at ≥1% mean relative abundance in healthy dogs at baseline (day 0). Mean percentages and standard deviation of relatively abundant bacteria, annotated to the level of phylum, family, and genus, are represented.
(DOCX)

S3 Table Alpha diversity averages for control eyes and treatment eyes over time measured at 13,338 sequences per sample. (DOCX)

S1 Fig Rarefaction analysis of 16S rRNA gene sequences from healthy dogs, comparing treatment and control eyes. Lines represent the mean of each group for all three time points sampled.
(TIF)

S2 Fig Principal coordinate analysis plot (PCoA) of unweighted UniFrac distance matrices of left (OS) and right (OD) eyes from 13 healthy dogs at baseline. Clustering was not observed between left and right eyes of individual dogs. Based on ANOSIM, pairwise comparisons between dogs were not signifant (Unweighted UniFrac, 999 permutations: mean R = -0.008, median R = -0.008, SD = 0.111, p > 0.05 (ranging from 0.333–1).
(TIF)

S3 Fig Principal coordinate analysis plot (PCoA) of unweighted UniFrac distance matrices of both eyes (n = 26) from healthy dogs (n = 13) at baseline. Clustering was not observed between dogs with shared households. Based on ANOSIM, pairwise comparison between households was not significant (Unweighted Unifrac, R = 0.313, p > 0.05).
(TIF)

S4 Fig Bacterial family composition of individual control eyes (n = 13) over time. Bars represent mean relative abundance of all taxa present in ≥ 6/13 eyes at each time point.
(TIF)

S5 Fig Bacterial family composition of individual treatment eyes (n = 13) over time. Bars represent mean relative abundance of all taxa present in ≥ 6/13 eyes at each time point.
(TIF)
