# Supplementary material for: Evaluation of the bacterial ocular surface microbiome in ophthalmologically normal dogs prior to and following treatment with topical neomycin-polymyxin-bacitracin
Source: PLoS One. 2020 Jun 9;15(6):e0234313. doi: 10.1371/journal.pone.0234313 (PMC7282667; doi:10.1371/journal.pone.0234313)
Supplement: S1 Checklist — (PDF) [file pone.0234313.s001.pdf]

Canine Ocular Microbiome Project

Patient ID	Clinician Information

☐ COMPLETE OPHTHALMIC EXAM AND CONJUNCTIVAL SWABS (05/21/18)

☐ REPEAT CONJUNCTIVAL SWABS (05/28/18 @ 8am) *Return Treatment Sheet*

☐ REPEAT CONJUNCTIVAL SWABS (06/25/18 @ 8am)

TREATMENT SHEET

NeoPolyBac: Give a ¼ inch strip in the RIGHT EYE three times daily for 1 week.

	Morning/Before Work	Afternoon/After Work	Evening/Before Bed
**05/21/18**		☐ First dose	☐
**05/22/18**	☐	☐	☐
**05/23/18**	☐	☐	☐
**05/24/18**	☐	☐	☐
**05/25/18**	☐	☐	☐
**05/26/18**	☐	☐	☐
**05/27/18**	☐	☐	☐
**05/28/18**	☐ Last dose		

COMMENTS

Investigator Signature: ____ Date: _______________
