# Supplementary material for: Evaluation of the bacterial ocular surface microbiome in ophthalmologically normal dogs prior to and following treatment with topical neomycin-polymyxin-bacitracin
Source: PLoS One. 2020 Jun 9;15(6):e0234313. doi: 10.1371/journal.pone.0234313 (PMC7282667; doi:10.1371/journal.pone.0234313)
Supplement: S1 Table — (DOCX) [file pone.0234313.s002.docx]

**S1 Table. Alpha diversity averages for eyes at baseline (day 0) measured at 13,338 sequences per sample.**

|  | **Control Eyes** | **Treatment Eyes** | **^*^P-value** |
| --- | --- | --- | --- |
| **Observed ASVs** | 108.30 ± 61.98 | 100.70 ± 52.35 | 0.850 |
| **Shannon** | 6.01 ± 0.96 | 5.84 ± 1.13 | 0.762 |
| **Chao1** | 108.30 ± 61.98 | 100.70 ± 52.35 | 0.850 |

Values represent averages with standard deviations. *P-values determined by Wilcoxon matched-pairs signed-ranks test with significance level < 0.05.
