# Supplementary material for: Evaluation of the bacterial ocular surface microbiome in ophthalmologically normal dogs prior to and following treatment with topical neomycin-polymyxin-bacitracin
Source: PLoS One. 2020 Jun 9;15(6):e0234313. doi: 10.1371/journal.pone.0234313 (PMC7282667; doi:10.1371/journal.pone.0234313)
Supplement: S2 Table — Mean percentages and standard deviation of relatively abundant bacteria, annotated to the level of phylum, family, and genus, are represented. (DOCX) [file pone.0234313.s003.docx]

**S2 Table. Taxa present at** $\boldsymbol{\geq}$**1% mean relative abundance in healthy dogs at baseline (day 0).** Mean percentages and standard deviation of relatively abundant bacteria, annotated to the level of phylum, family, and genus, are represented.

| **Taxon** | **Healthy Dogs at Baseline** | | |
| --- | --- | --- | --- |
| **Phylum**  Family  *Genus* | Mean % | SD % | Number of eyes with positive detection (n=26) |
| **Proteobacteria** | 49.7 | 20.6 | 26 |
| Pseudomonadaceae | 13.2 | 25.0 | 26 |
| *Unclassified Pseudomonadaceae* | 9.3 | 24.2 | 22 |
| *Pseudomonas* spp. | 3.3 | 4.9 | 23 |
| Pasteurellaceae | 6.9 | 12.2 | 23 |
| *Unclassified Pasteurellaceae* | 5.9 | 10.8 | 23 |
| Enterobacteriaceae | 3.9 | 5.8 | 23 |
| *Unclassified Enterobacteriaceae* | 3.2 | 4.7 | 23 |
| Neisseriaceae | 3.5 | 4.7 | 19 |
| *Unclassified Neisseriaceae* | 1.9 | 2.9 | 15 |
| Moraxellaceae | 3.0 | 3.4 | 22 |
| *Moraxella* spp. | 1.6 | 3.0 | 15 |
| *Acinetobacter* spp. | 1.0 | 1.0 | 15 |
| Sphingomonadaceae | 2.4 | 2.5 | 23 |
| *Sphingomonas* spp. | 1.0 | 1.5 | 17 |
| *Kaistobacter* spp. | 1.0 | 1.4 | 13 |
| Oxalobacteraceae | 2.3 | 1.9 | 22 |
| *Unclassified Oxalobacteraceae* | 1.4 | 1.6 | 18 |
| Rhodobacteraceae | 2.0 | 2.9 | 21 |
| *Paracoccus* spp. | 1.4 | 2.8 | 19 |
| Methylobacteriaceae | 1.9 | 8.2 | 17 |
| *Methylobacterium* spp. | 1.9 | 8.2 | 17 |
| Comamonadaceae | 1.7 | 1.2 | 23 |
| *Delftia* spp. | 1.0 | 1.0 | 15 |
| Xanthomonadaceae | 1.5 | 1.4 | 23 |
| *Unclassified Xanthomonadaceae* | 1.0 | 1.1 | 12 |
| Bradyrhizobiaceae | 1.3 | 1.4 | 19 |
| *Unclassified Bradyrhizobiaceae* | 1.1 | 1.4 | 19 |
| Burkholderiaceae | 1.0 | 1.0 | 19 |
| *Burkholderia* spp. | 1.0 | 1.0 | 15 |
| **Actinobacteria** | 25.5 | 17.9 | 26 |
| Micrococcaceae | 12.0 | 14.3 | 25 |
| *Unclassified Micrococcaceae* | 11.0 | 14.5 | 23 |
| Microbacteriaceae | 5.2 | 4.8 | 26 |
| *Salinibacterium* spp. | 4.7 | 4.8 | 26 |
| Corynebacteriaceae | 3.3 | 3.7 | 23 |
| *Corynebacterium* spp. | 3.3 | 3.7 | 23 |
| Nocardioidaceae | 1.0 | 1.0 | 17 |
| Intrasporangiaceae | 1.0 | 1.2 | 12 |
| **Firmicutes** | 12.0 | 7.2 | 26 |
| Staphylococcaceae | 2.3 | 2.6 | 21 |
| *Staphylococcus* spp. | 2.0 | 2.3 | 20 |
| Bacillaceae | 1.5 | 1.5 | 20 |
| *Bacillus* spp. | 1.2 | 1.2 | 17 |
| Bradyrhizobiaceae | 1.3 | 1.4 | 19 |
| Clostridiaceae | 1.3 | 2.9 | 11 |
| *Clostridium* spp. | 1.0 | 2.1 | 10 |
| Streptococcaceae | 1.2 | 1.4 | 17 |
| *Streptococcus* spp. | 1.2 | 1.4 | 17 |
| Lachnospiraceae | 1.0 | 1.0 | 15 |
| **Bacteroidetes** | 7.5 | 6.0 | 26 |
| Porphyromonadaceae | 2.4 | 3.3 | 16 |
| *Porphyromonas* spp. | 2.2 | 3.1 | 15 |
| Bacteroidaceae | 1.2 | 2.4 | 14 |
| *Bacteroides* spp. | 1.2 | 2.4 | 14 |
| Paraprevotellaceae | 1.0 | 1.0 | 14 |
| *Prevotella* spp. | 1.0 | 1.0 | 13 |
| Cytophagaceae | 1.0 | 1.0 | 12 |
| Sphingobacteriaceae | 1.0 | 1.0 | 12 |
| Weeksellaceae | 1.0 | 1.3 | 17 |
| **Fusobacteria** | 1.4 | 1.9 | 14 |
| Fusobacteriaceae | 1.1 | 1.6 | 14 |
| *Fusobacterium* spp. | 1.1 | 1.6 | 14 |
