# Supplementary material for: Evaluation of the bacterial ocular surface microbiome in ophthalmologically normal dogs prior to and following treatment with topical neomycin-polymyxin-bacitracin
Source: PLoS One. 2020 Jun 9;15(6):e0234313. doi: 10.1371/journal.pone.0234313 (PMC7282667; doi:10.1371/journal.pone.0234313)
Supplement: S3 Table — (DOCX) [file pone.0234313.s004.docx]

**S3 Table. Alpha diversity averages for control eyes and treatment eyes over time measured at 13,338 sequences per sample.**

|  | **Day 0**  **(Baseline)** | **Day 7** | **Day 35** | **^*^P-value** |
| --- | --- | --- | --- | --- |
| **Control Eyes** | | | | |
| **Observed ASVs** | 108.30 ± 61.98 | 105.30 ± 46.53 | 107.5 ± 47.76 | 0.466 |
| **Shannon** | 6.01 ± 0.96 | 5.88 ± 0.93 | 6.07 ± 0.89 | 0.584 |
| **Chao1** | 108.30 ± 61.98 | 105.30 ± 46.53 | 107.5 ± 47.76 | 0.466 |
| **Treatment Eyes** | | | | |
| **Observed ASVs** | 100.70 ± 52.35 | 121.00 ± 54.62 | 100.20 ± 32.94 | 0.096 |
| **Shannon** | 5.84 ± 1.13 | 6.12 ± 1.21 | 6.01 ± 0.74 | 0.292 |
| **Chao1** | 100.70 ± 52.35 | 121.10 ± 54.80 | 100.30 ± 33.09 | 0.149 |

Values represent averages with standard deviations. *P-values determined by Friedman test and Dunn’s post-test with significance level < 0.05.
