# Supplementary figures and images for: Evaluation of the bacterial ocular surface microbiome in ophthalmologically normal dogs prior to and following treatment with topical neomycin-polymyxin-bacitracin
Source: PLoS One. 2020 Jun 9;15(6):e0234313. doi: 10.1371/journal.pone.0234313 (PMC7282667; doi:10.1371/journal.pone.0234313)

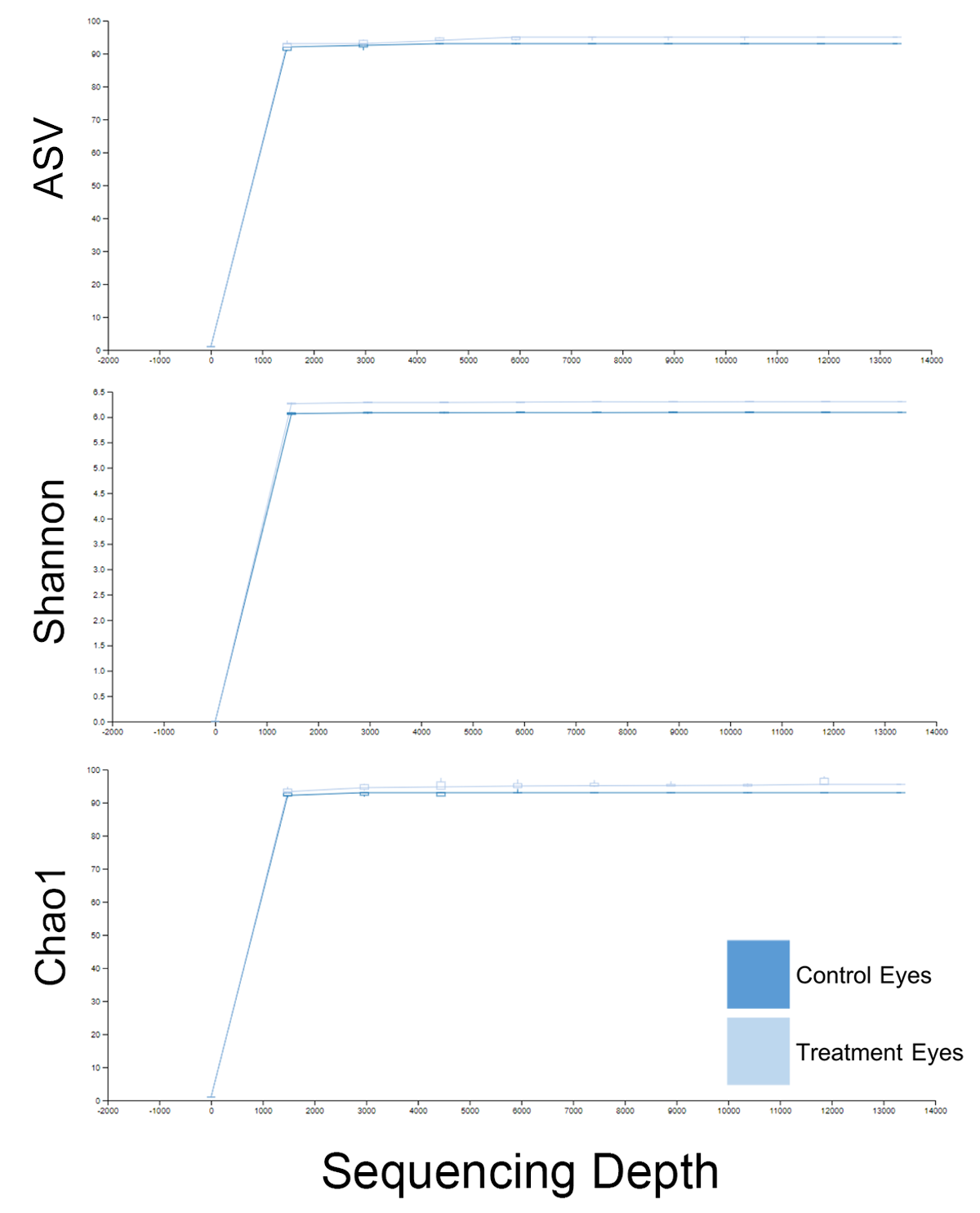

Supplement: S1 Fig — Lines represent the mean of each group for all three time points sampled. (TIF) [file pone.0234313.s005.tif]

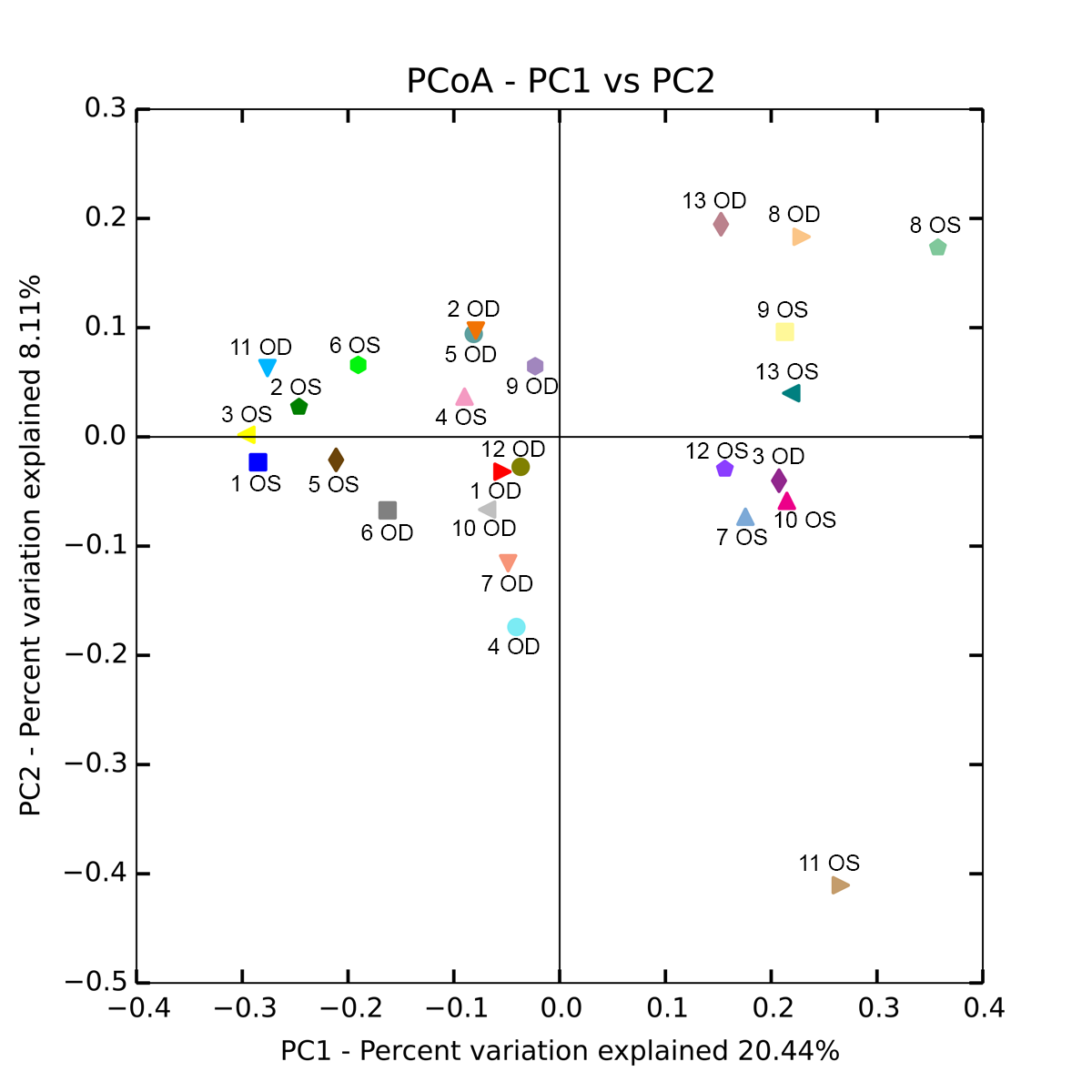

Supplement: S2 Fig — Clustering was not observed between left and right eyes of individual dogs. Based on ANOSIM, pairwise comparisons between dogs were not signifant (Unweighted UniFrac, 999 permutations: mean R = -0.008, median R = -0.008, SD = 0.111, p > 0.05 (ranging from 0.333–1). (TIF) [file pone.0234313.s006.tif]

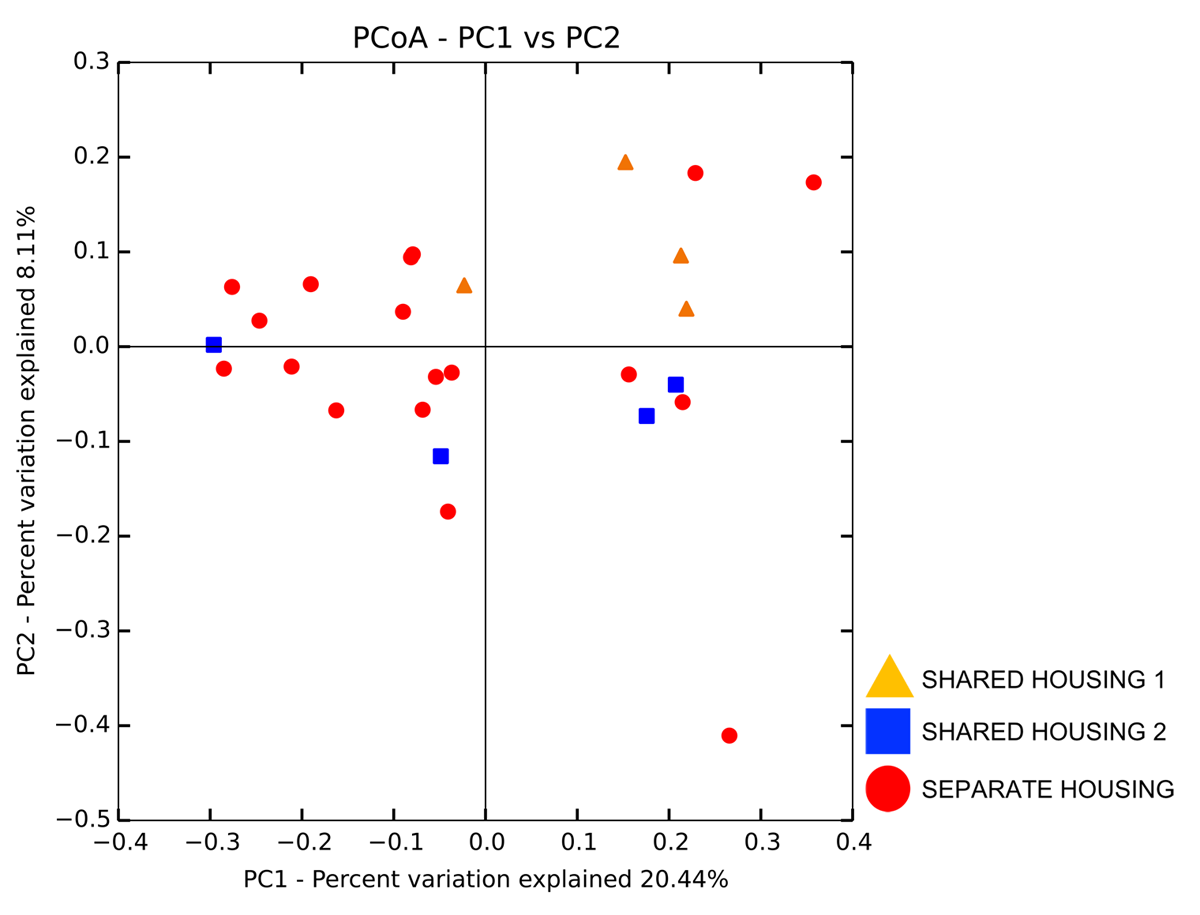

Supplement: S3 Fig — Clustering was not observed between dogs with shared households. Based on ANOSIM, pairwise comparison between households was not significant (Unweighted Unifrac, R = 0.313, p > 0.05). (TIF) [file pone.0234313.s007.tif]

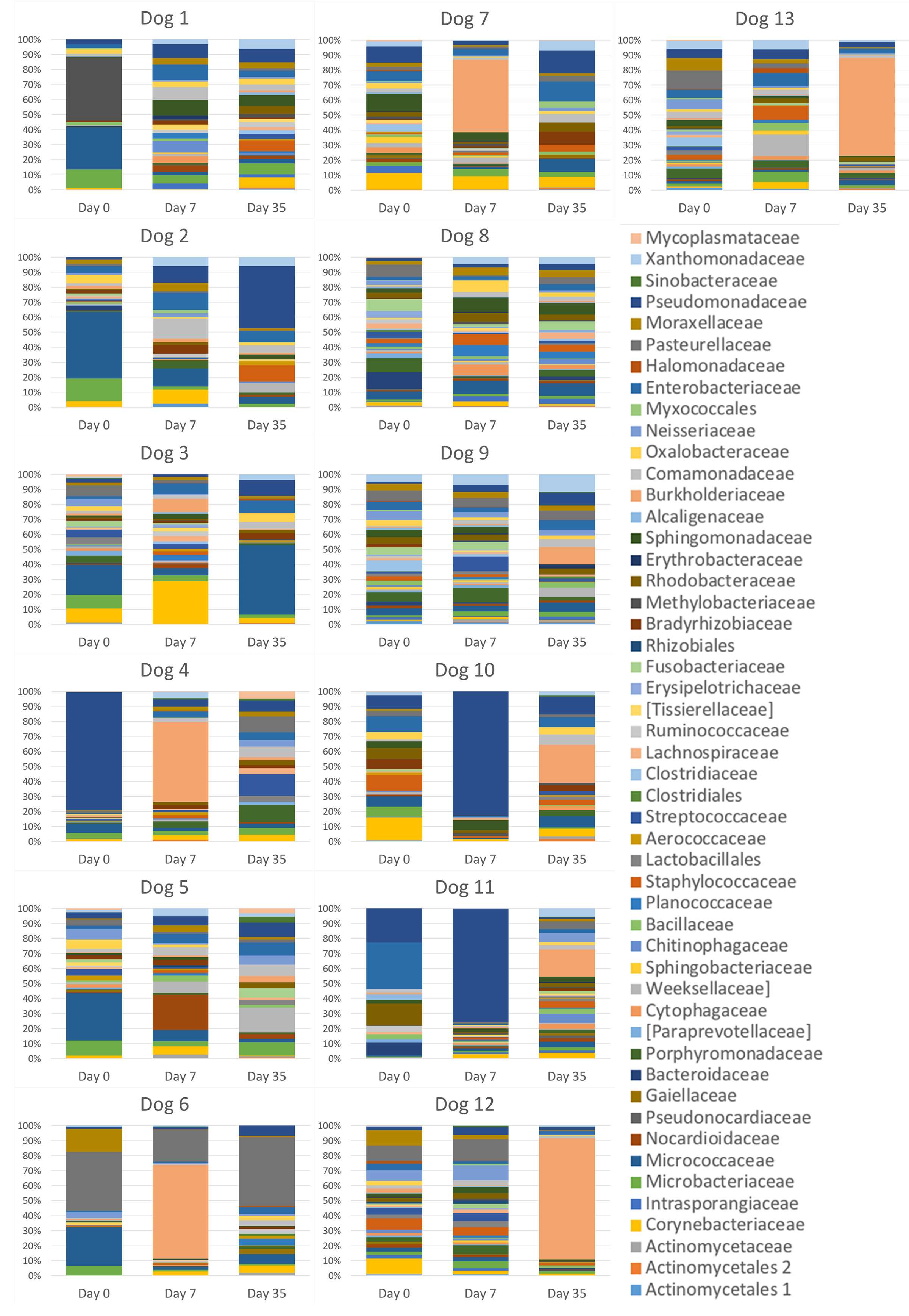

Supplement: S4 Fig — Bars represent mean relative abundance of all taxa present in ≥ 6/13 eyes at each time point. (TIF) [file pone.0234313.s008.tif]

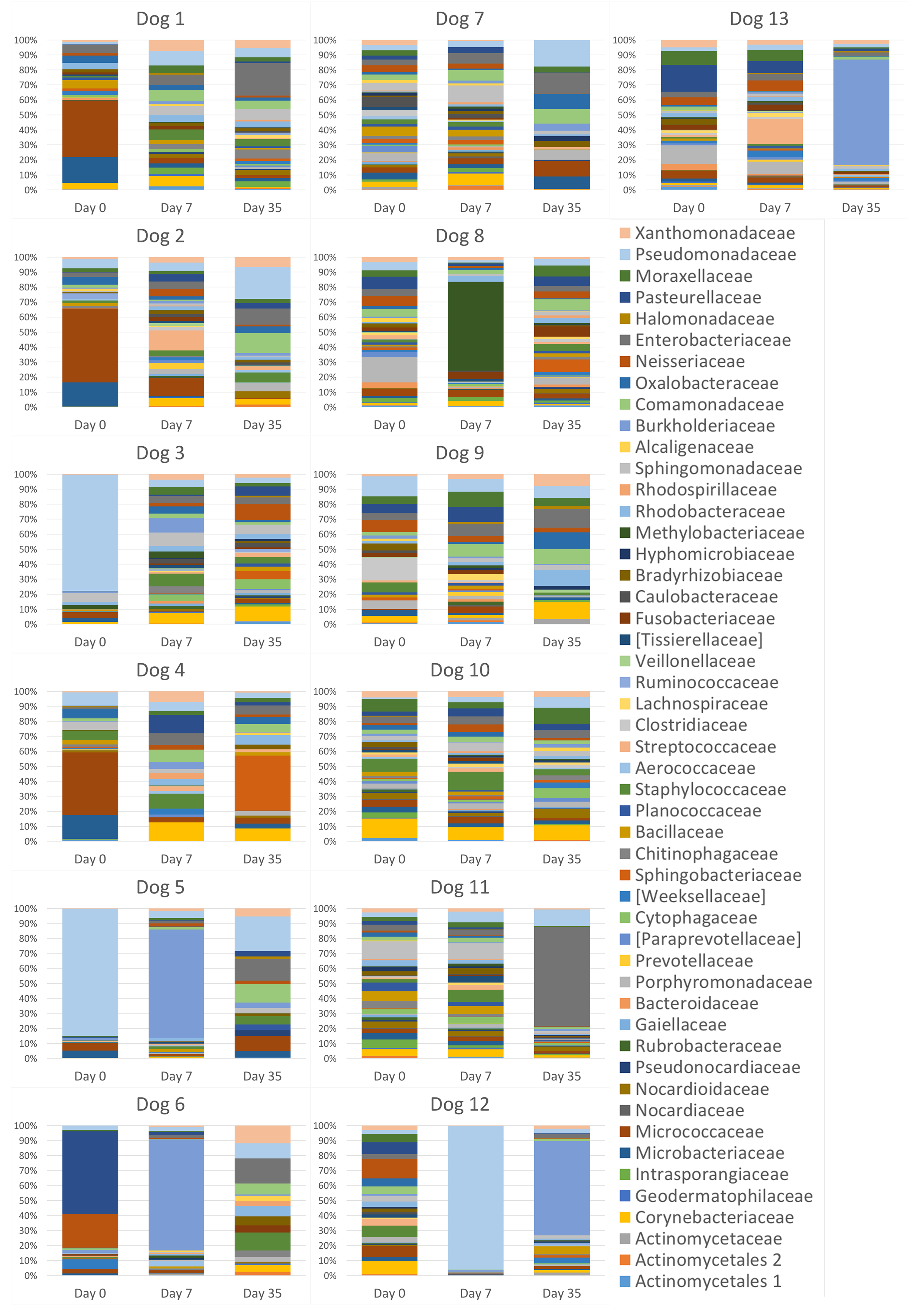

Supplement: S5 Fig — Bars represent mean relative abundance of all taxa present in ≥ 6/13 eyes at each time point. (TIF) [file pone.0234313.s009.tif]
